# A multi-scale semi-mechanistic CK/PD model for CAR T-cell therapy

**DOI:** 10.3389/fsysb.2024.1380018

**Published:** 2024-08-29

**Authors:** Sarah Minucci, Scott Gruver, Kalyanasundaram Subramanian, Marissa Renardy

**Affiliations:** ^1^ Applied BioMath, Concord, MA, United States; ^2^ Differentia Biotech, San Francisco, CA, United States

**Keywords:** CAR T-cell therapy, mechanistic modeling, quantitative systems pharmacology, sensitivity analysis, cellular kinetics

## Abstract

Chimeric antigen receptor T (CAR T) cell therapy has shown remarkable success in treating various leukemias and lymphomas. Cellular kinetic (CK) and pharmacodynamic (PD) behavior of CAR T cell therapy is distinct from other therapies due to its living nature. CAR T CK is typically characterized by an exponential expansion driven by target binding, fast initial decline (contraction), and slow long-term decline (persistence). Due to the dependence of CK on target binding, CK and PD of CAR T therapies are inherently and bidirectionally linked. In this work, we develop a semi-mechanistic model of CAR T CK/PD, incorporating molecular-scale binding, T cell dynamics with multiple phenotypes, and tumor growth and killing. We calibrate this model to published CK and PD data for a CD19-targeting CAR T cell therapy. Using sensitivity analysis, we explore variability in response due to patient- and drug-specific properties. We further explore the impact of tumor characteristics on CAR T-cell expansion and efficacy through individual- and population-level parameter scans.

## 1 Introduction

Chimeric antigen receptor (CAR) T-cells are T-cells engineered to produce CARs which recognize and bind to a tumor antigen. In CAR T-cell therapy, a patient’s T-cells are extracted and isolated, re-engineered to express a specific CAR, expanded *ex vivo*, and then infused back into the patient. Six such therapies have been approved for treating a variety of blood cancers (Chen et al., 2023). These therapies have been shown to produce long-lasting response and superior response rates to alternative treatments (Melenhorst et al., 2022; Sermer et al., 2020). As a result of the individualized nature of CAR T manufacturing, the contents of the dosed product will vary from patient to patient. Further, CAR T cellular kinetic behavior is distinct from other therapies due to its “living” nature; it is typically characterized by an exponential expansion, fast initial decline (contraction), and slow long-term decline (persistence). Additionally, cellular kinetics (CK) is not as well-studied as pharmacokinetics for more traditional drugs Chaudhury et al. (2020). Interactions between CAR T-cells and tumor cells are complex since tumor expansion has a significant impact on CAR T-cell expansion. Furthermore, much is still unknown about the workings of CAR T-cells in the body and there is not a standard monitoring process. Modeling can shed light on CAR-T cell CK/PD and inform future studies by mechanistically linking CAR T-cell doses to tumor cell growth and determining optimal drug properties to achieve efficacy and safety. Furthermore, patient characteristics can be incorporated into the model to provide individualized dose predictions and guide patient and indication selection.

Modeling and simulation has been used to understand CAR T-cell dynamics and efficacy (see, for example, reviews by Chaudhury et al. (2020); Nukala et al. (2021)) and the impact of preconditioning (Owens and Bozic, 2021). Until recently, the three distinct phases of CAR T cellular kinetics and the impact of different CD4^+^ and CD8^+^ T cell phenotypes had not been mechanistically described. Previous modeling work had captured CAR T cellular kinetics either empirically (Stein et al., 2019), mechanistically but without multiple phenotypes, (Singh et al., 2020), or mechanistically with effector/memory phenotypes but without separating CD4^+^ and CD8^+^ T cells (Hardiansyah and Ng, 2019). Recent work by Salem et al. (2023) has incorporated all of these features, developing a mechanistic model incorporating binding-driven CAR T-cell expansion and activity for multiple CD4^+^ and CD8^+^ T-cell phenotypes to match clinical data from multiple trials. Further analysis of such models will be useful to understand system behavior, inform engineering of CAR T-cells, and understand variability in patient populations. In particular, sensitivity analysis provides understanding of the key mechanisms driving expansion and efficacy.

Here, we present a semi-mechanistic cellular kinetic-pharmacodynamic (CK-PD) model for CAR T-cell therapy of B-cell non-Hodgkin lymphoma (NHL). Our model includes CD8^+^ and CD4^+^ naive, effector, and memory T-cell phenotypes, binding of CARs to their target antigen CD19, binding-driven activation and expansion of T-cells, T-cell death and conversion to memory cells, and binding-driven killing of B cells by CD8^+^ effector cells. We demonstrate the ability of the model to capture published human CAR T-cell CK and PD data, and perform sensitivity analysis to understand key model features and predict the impact of variability in patient, tumor, and drug characteristics.

## 2 Methods

### 2.1 Data

The model was informed by and benchmarked to published human CAR T-cellular kinetics, B cell percentage, and clinical response data from a phase I clinical trial with IM19 CAR T-cells for 13 relapsed or refractory NHL patients (Ying et al., 2021). The CK data and the B cell aplasia data were both digitized using WebPlotDigitizer (Rohatgi, 2022). Two days prior to CAR T-cell infusion, patients were pre-treated with fludarabine and cyclophosphamide for 3 days to deplete endogenous lymphocytes. IM19 CAR T-cells were dosed by weight at $3\times 10^{5}$ , $1\times 10^{6}$ , or $3\times 10^{6}$ cells per kg. The CD4:CD8 ratio of the infused CAR T-cells was reported for each of the patients.

### 2.2 Model structure

**FIGURE 1:**
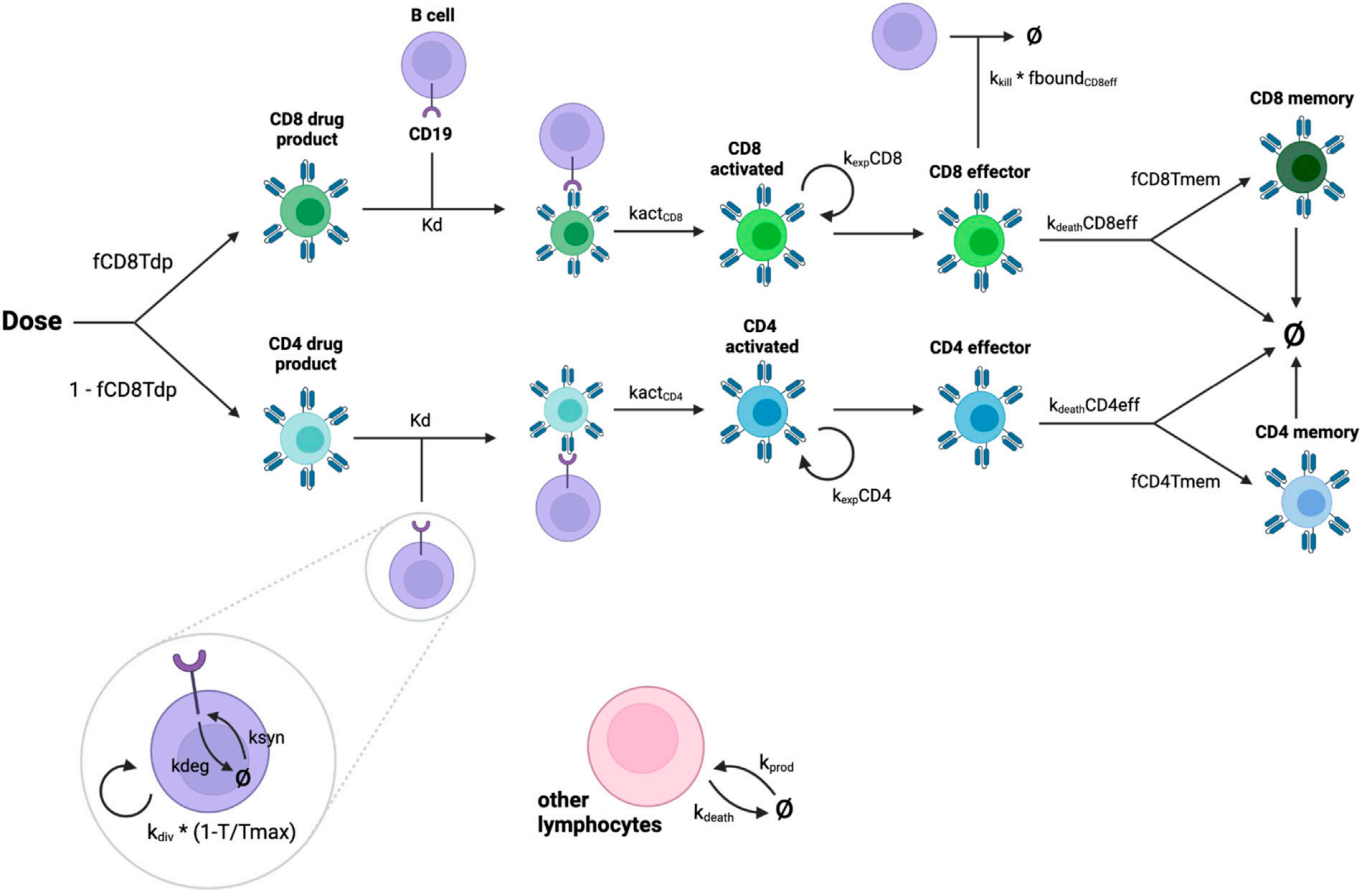
Diagram showing interactions represented in the model. CAR-T cells are dosed as part CD8^+^, part CD4^+^. Drug product cells are activated by binding to CD19 on malignant B cells. Activated cells replicate and become effector cells. CD8^+^ effector cells kill B cells. Effector CAR T-cells either die or become memory cells.

The model consists of a single compartment representing the blood. CAR T-cell and B cell populations and their corresponding receptor burdens are modeled explicitly. CAR T-cells, a fraction of which are CD8^+^ and the remainder CD4^+^, are dosed directly into the blood. All CAR T-cells are assumed to be naive at the time of dosing. CARs on both CD8^+^ and CD4^+^ CAR T-cells can bind to CD19. CD8^+^ and CD4^+^ naive CAR T-cells are activated at a rate proportional to the fraction of CAR that is bound to CD19. Activated CAR T-cells then proliferate and become effector cells. CD8^+^ effector CAR T-cells can then kill B cells at a rate proportional to the fraction of CARs on CD8^+^ effector T cells that are bound to CD19. We assume that CD4^+^ effector CAR T-cells do not kill B cells as we focus only on direct effects (Alizadeh et al., 2023). Effector CAR T-cells either die or become memory cells. Memory CAR T-cells have a longer lifespan than effector cells, but do not participate in B cell killing. The model is intended to describe the initial response to CAR T therapy and therefore does not include any mechanisms for re-activation of memory cells. A diagram of the model reactions is shown in Figure 1. A more detailed description of the model equations is given below, where all states are in units of nmol.

### 2.3 Cell state equations

Infused CAR T cells are dosed directly into the blood ( $T_{\inf}^{\mathrm{CD8}}\left(0\right)=fCD8T\times Dose$ , where fCD8T is the fraction of dosed CAR T-cells that are CD8^+^). These cells can then become activated at a rate $k_{\mathrm{act}}$ or die at a rate $k_{\mathrm{death,inf}}$ . Activated CAR T cells divide at a rate $k_{\mathrm{div}}=\left(2^{\mathrm{ndiv}}-1\right)/\tau$ to form effector cells at a rate $k_{\mathrm{diff}}=2^{\mathrm{ndiv}}/\tau$ , where ndiv is the average number of divisions per activated cell and τ is the division time. At a rate of $k_{\mathrm{death,eff}}$ , a fraction $f_{\mathrm{mem}}$ of effector cells become memory cells and the remainder die. Memory T cells die at a rate of $k_{\mathrm{death,mem}}$ . This leads to the following equation for CD8^+^ CAR T cells, and similarly for CD4^+^ CAR T cells.

$$\begin{array}{ll} \frac{dT_{\inf}^{\mathrm{CD8}}}{dt} & =-k_{\mathrm{act}}^{\mathrm{CD8}}T_{\inf}^{\mathrm{CD8}}-k_{\mathrm{death,inf}}^{\mathrm{CD8}}T_{\inf}^{\mathrm{CD8}}\\ \frac{dT_{\mathrm{act}}^{\mathrm{CD8}}}{dt} & =k_{\mathrm{act}}^{\mathrm{CD8}}T_{\inf}^{\mathrm{CD8}}+k_{\mathrm{div}}^{\mathrm{CD8}}T_{\mathrm{act}}^{\mathrm{CD8}}-k_{\mathrm{diff}}^{\mathrm{CD8}}T_{\mathrm{act}}^{\mathrm{CD8}}\\ \frac{dT_{\mathrm{eff}}^{\mathrm{CD8}}}{dt} & =k_{\mathrm{diff}}^{\mathrm{CD8}}T_{\mathrm{act}}^{\mathrm{CD8}}-k_{\mathrm{death,eff}}^{\mathrm{CD8}}T_{\mathrm{eff}}^{\mathrm{CD8}}\\ \frac{dT_{\mathrm{mem}}^{\mathrm{CD8}}}{dt} & =k_{\mathrm{death,eff}}^{\mathrm{CD8}}f_{\mathrm{mem}}^{\mathrm{CD8}}T_{\mathrm{eff}}^{\mathrm{CD8}}-k_{\mathrm{death,mem}}^{\mathrm{CD8}}T_{\mathrm{mem}}^{\mathrm{CD8}} \end{array}$$

Tumor cells are able to divide at a rate $k_{\mathrm{div}}^{\mathrm{tum}}$ and be killed by CD8^+^ effector CAR T cells at a rate $k_{\mathrm{kill}}=f_{\mathrm{bound}}k_{\mathrm{maxkill}}$ , where $f_{\mathrm{bound}}$ is the fraction of CD8^+^ CAR that is bound to CD19.

$$\frac{dTumor}{dt}=k_{\mathrm{div}}^{\mathrm{tum}}Tumor-k_{\mathrm{kill}}*Tumor*T_{\mathrm{eff}}^{\mathrm{CD8}}$$

Endogenous lymphocytes are produced at a zeroth order rate $k_{\mathrm{prod}}$ and die at a first order rate $k_{\mathrm{death,endo}}$ , resulting in the following equation.

$$\frac{dEndo}{dt}=k_{\mathrm{prod}}Endo-k_{\mathrm{death,endo}}Endo$$

### 2.4 Receptor equations

In addition to the cell-scale dynamics described above, molecular-scale dynamics are explicitly accounted for in the model. Receptor equations for CAR and CD19 are written such that the total receptor densities (CAR per T cell and CD19 per tumor cell) remain constant, as determined by a receptor per cell (RPC) parameter. Receptors are synthesized at a rate $k_{\mathrm{syn}}$ and internalized at a rate $k_{\mathrm{int}}$ . The equation for CD19 is written as follows, accounting for tumor cell division, synthesis and internalization of free CD19, binding/unbinding to CAR on different types of T cells, release from CAR:CD19 complex that is internalized with CAR, release from CAR:CD19 complex when a CAR T cell dies, and tumor cell death.

$$\begin{array}{ll} \frac{dmAg}{dt} & =k_{\mathrm{div}}^{\mathrm{tum}}*Tumor*RPC_{\mathrm{CD19}}/\left(N_{Av}/1e9\right)+k_{\mathrm{syn}}^{\mathrm{CD19}}Tumor-k_{\mathrm{int}}^{\mathrm{CD19}}CD19\\ & -\frac{k_{on}}{V}\left(CAR_{\inf}^{\mathrm{CD8}}+CAR_{\inf}^{\mathrm{CD4}}+CAR_{\mathrm{eff}}^{\mathrm{CD8}}+CAR_{\mathrm{eff}}^{\mathrm{CD4}}+CAR_{\mathrm{mem}}^{\mathrm{CD8}}+CAR_{\mathrm{mem}}^{\mathrm{CD4}}\right)CD19\\ & +k_{\mathrm{off}}\left(CAR_{\inf}^{\mathrm{CD8}}:CD19+CAR_{\inf}^{\mathrm{CD4}}:CD19+CAR_{\mathrm{eff}}^{\mathrm{CD8}}:CD19+CAR_{\mathrm{eff}}^{\mathrm{CD4}}:CD19+\right.\\ & \;\left.CAR_{\mathrm{mem}}^{\mathrm{CD8}}:CD19+CAR_{\mathrm{mem}}^{\mathrm{CD4}}:CD19\right)\\ & +k_{\mathrm{int}}^{\mathrm{CAR}}\left(CAR_{\inf}^{\mathrm{CD8}}:CD19+CAR_{\inf}^{\mathrm{CD4}}:CD19+CAR_{\mathrm{eff}}^{\mathrm{CD8}}:CD19+CAR_{\mathrm{eff}}^{\mathrm{CD4}}:CD19+\right.\\ & \;\left.CAR_{\mathrm{mem}}^{\mathrm{CD8}}:CD19+CAR_{\mathrm{mem}}^{\mathrm{CD4}}:CD19\right)\\ & +k_{\mathrm{death,inf}}^{\mathrm{CD8}}CAR_{\inf}^{\mathrm{CD8}}:CD19+k_{\mathrm{death,inf}}^{\mathrm{CD4}}CAR_{\inf}^{\mathrm{CD4}}:CD19+k_{\mathrm{death,eff}}^{\mathrm{CD8}}CAR_{\mathrm{eff}}^{\mathrm{CD8}}:CD19\\ & +k_{\mathrm{death,eff}}^{\mathrm{CD4}}CAR_{\mathrm{eff}}^{\mathrm{CD4}}:CD19+k_{\mathrm{death,mem}}^{\mathrm{CD8}}CAR_{\mathrm{mem}}^{\mathrm{CD8}}:CD19+k_{\mathrm{death,mem}}^{\mathrm{CD4}}CAR_{\mathrm{mem}}^{\mathrm{CD4}}:CD19\\ & -k_{\mathrm{kill}}T_{\mathrm{eff}}^{\mathrm{CD8}}CD19 \end{array}$$

CARs on infused CAR T cells undergo synthesis and internalization, binding/unbinding with CD19, conversion to an activated state, loss from cell death, and release from CAR:CD19 complex when a tumor cell dies. The equations for CD8^+^ infused CAR and CAR:CD19 complex are shown below; equations for CD4^+^ CAR are similar.

$$\begin{array}{ll} \frac{dCAR_{\inf}^{\mathrm{CD8}}}{dt} & =k_{\mathrm{syn}}^{\mathrm{CAR,CD8}}T_{\inf}^{\mathrm{CD8}}-k_{\mathrm{int}}^{\mathrm{CAR}}CAR_{\inf}^{\mathrm{CD8}}-\frac{k_{on}}{V}CAR_{\inf}^{\mathrm{CD8}}CD19\\ & \;+k_{\mathrm{off}}CAR_{\inf}^{\mathrm{CD8}}:CD19-k_{\mathrm{act}}^{\mathrm{CD8}}CAR_{\inf}^{\mathrm{CD8}}+k_{\mathrm{kill}}T_{\mathrm{eff}}^{\mathrm{CD8}}CAR_{\inf}^{\mathrm{CD8}}:CD19\\ & \;-k_{\mathrm{death,inf}}^{\mathrm{CD8}}CAR_{\inf}^{\mathrm{CD8}}\\ \frac{dCAR_{\inf}^{\mathrm{CD8}}:CD19}{dt} & =\frac{k_{on}}{V}CAR_{\inf}^{\mathrm{CD8}}CD19-k_{\mathrm{off}}CAR_{\inf}^{\mathrm{CD8}}:CD19-k_{\mathrm{int}}^{\mathrm{CAR}}CAR_{\inf}^{\mathrm{CD8}}:CD19\\ & \;-k_{\mathrm{act}}^{\mathrm{CD8}}CAR_{\inf}^{\mathrm{CD8}}:CD19-k_{\mathrm{kill}}T_{\mathrm{eff}}^{\mathrm{CD8}}CAR_{\inf}^{\mathrm{CD8}}:CD19\\ & \;-k_{\mathrm{death,inf}}^{\mathrm{CD8}}CAR_{\inf}^{\mathrm{CD8}}:CD19 \end{array}$$

Since activated CAR T cells in the model are an intermediate state for the purposes of expansion and do not interact with the tumor, we do not include binding of activated CARs to CD19 in the model. CARs on activated CAR T cells follow the cellular kinetics (i.e., activation of infused CAR, division, and differentiation) so that CAR per T cell remains constant.

$$\frac{dCAR_{\mathrm{act}}^{\mathrm{CD8}}}{dt}=k_{\mathrm{act}}^{\mathrm{CD8}}\left(CAR_{\inf}^{\mathrm{CD8}}+CAR_{\inf}^{\mathrm{CD8}}:mAg\right)+k_{\mathrm{div}}^{\mathrm{CD8}}CAR_{\mathrm{act}}^{\mathrm{CD8}}-k_{\mathrm{diff}}^{\mathrm{CD8}}CAR_{\mathrm{act}}^{\mathrm{CD8}}$$

CARs on effector and memory CAR T cells are synthesized and internalized, bind/unbind with CD19, undergo effector-to-memory conversion, are lost through T cell death, and are released from CAR:CD19 complex when a tumor cell dies. These equations are given below for CD8^+^ effector and memory CAR; equations for CD4^+^ CARs are similar.

$$\begin{array}{ll} \frac{dCAR_{\mathrm{eff}}^{\mathrm{CD8}}}{dt} & =k_{\mathrm{diff}}^{\mathrm{CD8}}CAR_{\mathrm{act}}^{\mathrm{CD8}}+k_{\mathrm{syn}}^{\mathrm{CAR,CD8}}T_{\mathrm{eff}}^{\mathrm{CD8}}-k_{\mathrm{int}}^{\mathrm{CAR}}CAR_{\mathrm{eff}}^{\mathrm{CD8}}-\frac{k_{on}}{V}CAR_{\mathrm{eff}}^{\mathrm{CD8}}CD19\\ & \;+k_{\mathrm{off}}CAR_{\mathrm{eff}}^{\mathrm{CD8}}:CD19+k_{\mathrm{kill}}T_{\mathrm{eff}}^{\mathrm{CD8}}CAR_{\mathrm{eff}}^{\mathrm{CD8}}:CD19-k_{\mathrm{death,eff}}^{\mathrm{CD8}}CAR_{\mathrm{eff}}^{\mathrm{CD8}}\\ \frac{dCAR_{\mathrm{eff}}^{\mathrm{CD8}}:CD19}{dt} & =\frac{k_{on}}{V}CAR_{\mathrm{eff}}^{\mathrm{CD8}}CD19-k_{\mathrm{off}}CAR_{\mathrm{eff}}^{\mathrm{CD8}}:CD19-k_{\mathrm{int}}^{\mathrm{CAR}}CAR_{\mathrm{eff}}^{\mathrm{CD8}}:CD19\\ & \;-k_{\mathrm{kill}}T_{\mathrm{eff}}^{\mathrm{CD8}}CAR_{\mathrm{eff}}^{\mathrm{CD8}}:CD19\\ \frac{dCAR_{\mathrm{mem}}^{\mathrm{CD8}}}{dt} & =k_{\mathrm{syn}}^{\mathrm{CAR,CD8}}T_{\mathrm{mem}}^{\mathrm{CD8}}-k_{\mathrm{int}}^{\mathrm{CAR}}CAR_{\mathrm{mem}}^{\mathrm{CD8}}-\frac{k_{on}}{V}CAR_{\mathrm{mem}}^{\mathrm{CD8}}CD19\\ & \;+k_{\mathrm{off}}CAR_{\mathrm{mem}}^{\mathrm{CD8}}:CD19+k_{\mathrm{kill}}T_{\mathrm{eff}}^{\mathrm{CD8}}CAR_{\mathrm{mem}}^{\mathrm{CD8}}:CD19\\ & \;+f_{\mathrm{mem}}^{\mathrm{CD8}}k_{\mathrm{death,eff}}^{\mathrm{CD8}}CAR_{\mathrm{eff}}^{\mathrm{CD8}}-k_{\mathrm{death,mem}}^{\mathrm{CD8}}CAR_{\mathrm{mem}}^{\mathrm{CD8}}\\ \frac{dCAR_{\mathrm{mem}}^{\mathrm{CD8}}:CD19}{dt} & =\frac{k_{on}}{V}CAR_{\mathrm{mem}}^{\mathrm{CD8}}CD19-k_{\mathrm{off}}CAR_{\mathrm{mem}}^{\mathrm{CD8}}:CD19-k_{\mathrm{int}}^{\mathrm{CAR}}CAR_{\mathrm{mem}}^{\mathrm{CD8}}:CD19\\ & \;-k_{\mathrm{kill}}T_{\mathrm{eff}}^{\mathrm{CD8}}CAR_{\mathrm{mem}}^{\mathrm{CD8}}:CD19 \end{array}$$

### 2.5 Model parameterization

Values for the majority of the model parameters were inferred from literature as described below. The rest of the parameters were fit to individual patient data from Ying et al. (2021), described below.

#### 2.3.1 Patient and tumor properties

The total blood volume was estimated to be 5L, based on the average human adult (Sharma and Sharma, 2018). The concentration of endogenous lymphocytes was assumed to be $10^{9}$ per L. Endogenous lymphocytes were estimated to have an average lifespan of 30 days based on a steady-state assumption and benchmarking to observed T-cell recovery following autologous transplant (Hakim et al., 2005). We assume that 90% of endogenous lymphocytes are depleted by chemotherapy pretreatment prior to CAR T-cell infusion (Ying et al., 2019). The carrying capacity for the number of tumor cells was estimated to be $7\times 10^{12}$ based on the maximum tumor volume reported in Press et al. (Press et al., 1993), assuming an average cell diameter of 10 μ m (Das et al., 1991) and dividing the tumor volume by average cell volume to obtain a maximum number of cells. CD19 expression was estimated to be 5,000 receptors per B cell based on published values for patients with different types of lymphoma (D’Arena et al., 2000; Malik-Chaudhry et al., 2021; Spiegel et al., 2021). The internalization half-life of CD19 was estimated to be 4 h; published data indicates the internalization half-life can be as fast as 30 min in human B-cell lymphoma cell lines (Du et al., 2008) but as slow as 12+ hours in B-cell chronic lymphocytic leukemia patient samples (Sieber et al., 2003).

#### 2.3.2 CAR T-cell properties

The CAR internalization half-life was estimated to be 6 h based on *in vitro* measurements for other CD19-targeting CAR T-cells Li et al. (2020). The mean activation time (that is, the time between binding to antigen and the start of cell proliferation) was estimated to be 18 h for CD8^+^ CAR T-cells (Henrickson et al., 2008; Cui and Kaech, 2010) and 36 h for CD4^+^ CAR T-cells (Kaech et al., 2002). Average lifespans for memory CAR T-cells were estimated to be 180 days for CD8^+^ and 240 days for CD4^+^ (Borghans et al., 2018). The CD4:CD8 ratio of the CAR T-cells for each patient were taken from Ying et al. (2021), and all infused CAR T-cells were assumed to be viable. We assumed expression levels of 12,700 CARs per T-cell for both CD8^+^ and CD4^+^ cells based on a published average estimate for a HMW-MAA-specific CAR on CD8^+^ T cells (Anikeeva et al., 2021). We assumed that CARs bind to CD19 with an affinity of 1 nM based on reported affinities for high affinity CAR T variants (Jayaraman et al., 2020), with a binding on-rate of 0.001/nM/s.

Remaining model parameters, namely, the number of divisions per T-cell, time per T-cell division, drug product and effector cell lifespan, memory cell fraction, and initial tumor burden, were fit to data as described in the following subsection.

#### 2.3.3 Calibration and benchmarking

Considerable variability in CAR T-cell expansion and efficacy is present in the data. To describe individual variability in CK, the following parameters were fit to individual CK trajectories: initial tumor burden, the number of divisions for activated T cells, and the fraction of effector cells that become memory cells. The time per T cell division and drug product and effector cell life spans were fit globally to all patient data. Optimization was performed using a Python-based trust region optimization method. Additionally, the percentage of B cells out of total cells in the model was calibrated to B cell aplasia data by tuning the number of endogenous lymphocytes in the model within a small, biologically reasonable range such that the mean and range of model outputs captured the general trend observed in the data. The rate of tumor cell division was also tuned to match the observed rebound in B cell aplasia data.

### 2.4 Model simulation and analysis

The model was implemented and simulations were performed with Applied BioMath’s proprietary QSP modeling platform. Analysis and plotting were performed with Python version 3.11.8.

Global sensitivity analysis (GSA) was evaluated using two methods: Sobol indices estimated via the Fourier Amplitude Sensitivity Test (FAST), implemented using SALib (Herman and Usher, 2017; Iwanaga et al., 2022), and partial rank correlation coefficients (PRCC), implemented using Pingouin (Vallat, 2018).

In the GSA, model parameters for which we had individual data or fitted values (body weight, fraction of CD8^+^ CAR T cells, initial tumor burden, number of CAR T cell divisions, and fraction of memory cells) were varied across the full range of individual values. Where possible, published ranges for individual parameters were used. CD19 expression was varied from 1,500 to 16,825 receptors per cell based on a published range for mantle cell lymphoma (D’Arena et al., 2000). Tumor doubling time was varied from 24 h to 30 days, based on the range reported in Roesch et al. (2014). Binding affinity was varied from 0.32 to 14.3 nM based on the range of values for CD19 CARs reported in Jayaraman et al. (2020). Remaining model parameters were varied 2-fold up and down nominal values. All parameters were sampled from a log-uniform distribution within their respective ranges, with a sample size of 5,000. Simulations were initialized with a $10^{6}$ cells/kg dose. Model outputs considered in the sensitivity analysis were the peak concentration of CAR T-cells (Cmax) and the tumor burden at day 20.

To explore temporal dynamics and explore the impact of tumor characteristics, we performed one-at-a-time scans of tumor division time and CD19 expression per cell. The model was simulated for specific patients as well as for the full patient population using different values of these parameters, while keeping other model parameters fixed.

## 3 Results

### 3.1 CK fitting and PD benchmarking

**FIGURE 2:**
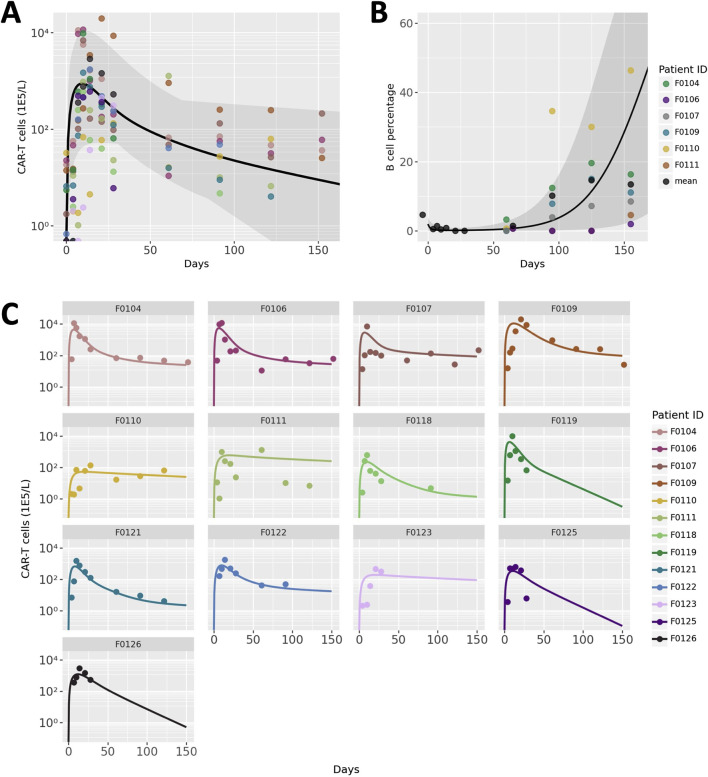
Model calibration and benchmarking results. **(A)** Patient population simulations for CAR T-cell CK. Black line indicates average model fit and shaded region represent the full range of individual trajectories. Points represent data, with colors representing different patients. **(B)** Patient population simulations for B cell aplasia. **(C)** Individual patient CAR T-cell CK data and simulations. Each panel represents an individual patient, the ID of which is labeled at the top of each panel.

CAR T-cell trajectories vary widely from patient to patient. Our model was developed and calibrated to capture the typical phases of CAR T-cell CK as well as the variability between patients through fitting a combination of patient-specific and global parameter values. Results of optimization of CAR T-cell concentration to clinical data are shown in Figure 2. Figure 2A shows the average trajectory and full range across all 13 patients and Figure 2C shows each fitted patient simulation and data. The model adequately describes the overall behavior of the data despite the significant variability between patients as well as within each patient data set. The full table of final parameter values can be found in the Supplementary Material.

Endogenous lymphocyte concentration and tumor doubling time were hand-tuned to a small degree to match measurements of B cells as a percentage of total lymphocytes, a measure of the efficacy of the CAR T-cells. Due to challenges with digitization, B cell aplasia data from only 6 of the 13 patients were distinguishable and are shown in Figure 2. The average and range of model simulations for all patient parameterizations capture the general trend of the data well and spans the variability between patients.

### 3.2 Sensitivity analysis

**FIGURE 3:**
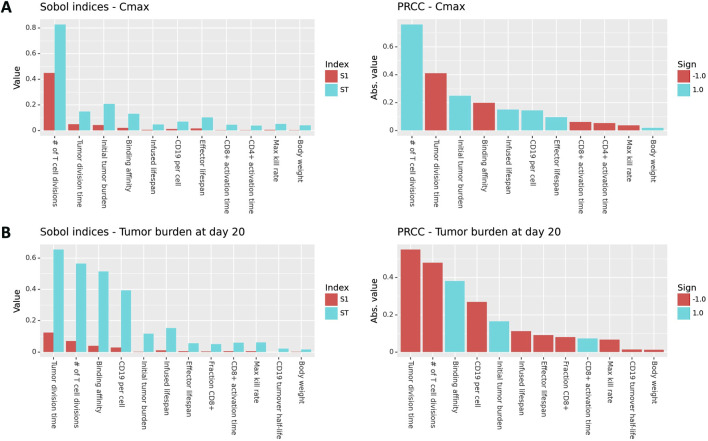
Global sensitivity results (Sobol indices and PRCC) for Cmax and tumor burden at day 20 post-treatment. Only parameters with a *p*-value less than 0.05 and that rank in the top ten for at least one measure of sensitivity are shown.

To explore the impact of model parameters on Cmax and efficacy, we performed Global Sensitivity Analysis (GSA). Results for Cmax are shown in Figure 3A. Sobol indices (first order and total order) and PRCC values are shown for all model parameters that had a *p*-value less than 0.05 and ranked in the top ten parameters for at least one measure of global sensitivity. The number of CAR T-cell divisions upon activation contributes to more than 80% of the variability in Cmax, which is a far greater contribution than any of the other parameters. The next most influential parameters are tumor growth rate, initial tumor burden, and mAb-CD19 binding affinity, which drive expansion through CAR-antigen interactions. CAR T cell life spans and CD19 expression are also influential. The ordering of parameters is roughly consistent between first order Sobol index, total order Sobol index, and PRCC. However, total order Sobol indices are generally at least two-fold larger than first order Sobol indices, indicating that there are interactions between parameters.

GSA results for tumor burden at day 20 are shown in Figure 3B. The most influential parameters are the tumor division time, number of T cell divisions, binding affinity, initial tumor burden, and CD19 expression is also influential. Tumor- and binding-related parameters are comparably influential on efficacy as the number of T cell divisions. This is in contrast to the results for Cmax, where the number of T cell divisions was by far the most influential parameter. This indicates that while expansion and efficacy are often correlated, patient properties such as tumor growth rate, initial tumor burden, and CD19 expression are more important for driving efficacy than they are for driving expansion. This is because CAR-CD19 interactions are required for both expansion and tumor cell killing.

### 3.3 Effects of tumor properties on CAR T expansion and efficacy

**FIGURE 4:**
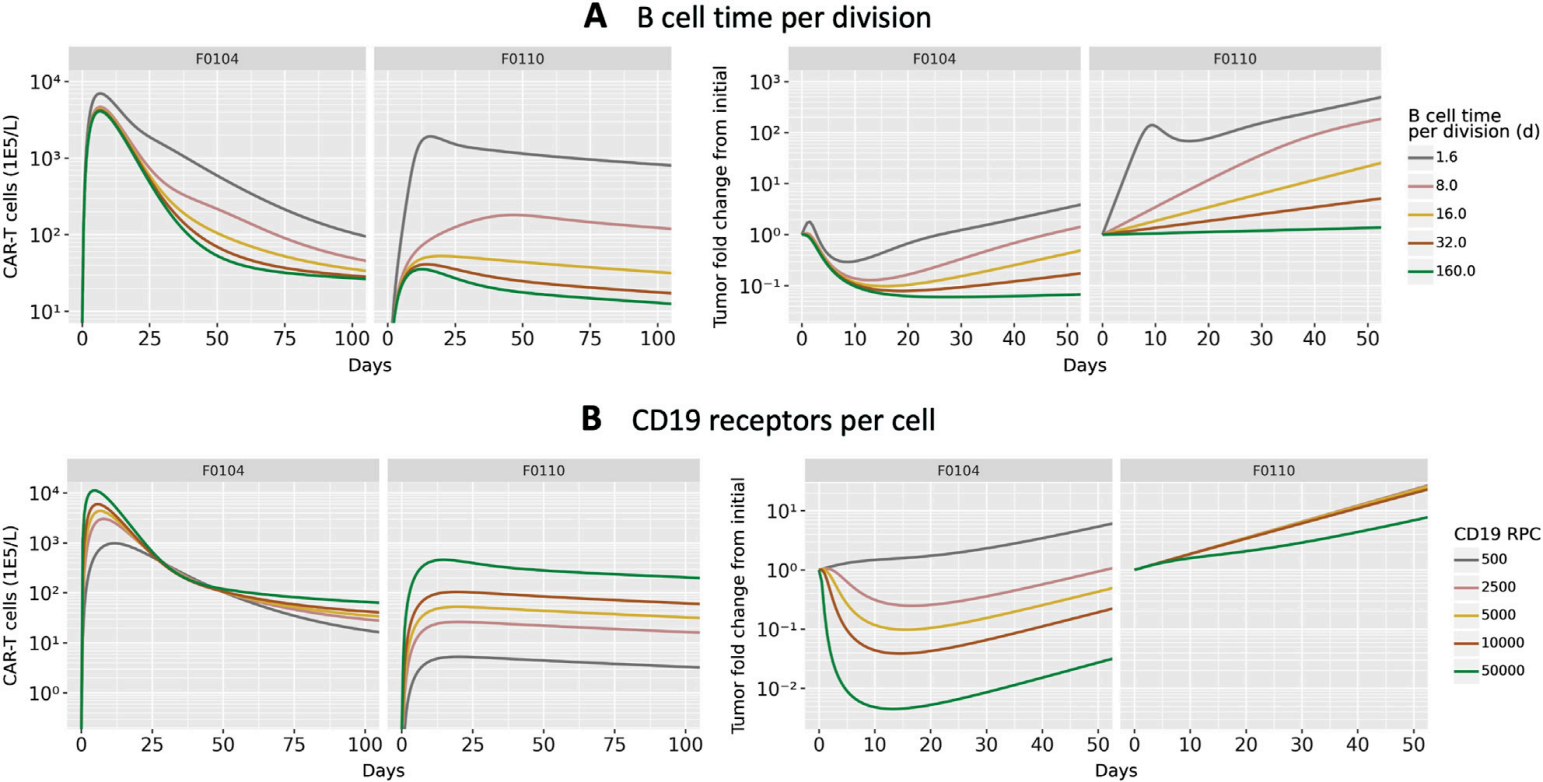
Results of scanning key model parameters representing patient characteristics for two individual patient parameterization. Parameters were scanned up to ∼ 10x above and below their nominal parameterization for patient parameterizations F0104 and F0110. Simulations of CAR T-cell concentration and total B cell fold change from initial are shown for scans of **(A)** B cell time per division and **(B)** CD19 receptors per cell.

To investigate potential mechanisms related to patient to patient variability in response, we evaluated the effects of B cell division time and CD19 expression on B cells. These two parameters, which were informed by literature and not varied in Figure 2C were shown to be influential parameters by the GSA. We first focus on two patient parameterizations (F0104 and F0110) which had distinct CK and tumor growth profiles. F0104 has a typical CK profile consisting of expansion, contraction, and persistence, paired with a clear reduction in tumor growth, while F0110 had continued tumor growth and less defined expansion and contraction phases. Figure 4 shows simulations of CAR T-cell concentration and tumor dynamics for these two patients, scanning over both parameters. Parameters are varied 10-fold up and down from nominal values to explore a wide range of system behaviors.

Scanning over B cell division time, shown in Figure 4A, revealed qualitatively different behavior between the two patients. For patient F0104, the model predicts that a B cell division time corresponds to a more gradual contraction, resulting in a greater concentration of CAR T-cells over time. The division time does not significantly impact the Cmax. However, for patient F0110, the slope of the contraction phase is relatively consistent across division times but the Cmax increases with faster division times. For both patients, the greater expansion of CAR T-cells is not sufficient to reverse tumor cell growth. A faster B cell division time results in more tumor growth regardless of CAR T-cell concentration for the parameter range scanned. Within the first 10–15 days, there is an acute reduction in tumor cells in response to initial CAR T-cell expansion for the fastest tumor cell division time, 1.6 days. However, this effect is transient and the faster B cell division time results in faster rebound of the tumor. For the slower tumor cell division times, CAR T expansion does reduce the tumor size; this combined with the generally slower tumor growth results in slow tumor growth in the longer term.

Figure 4B shows the results of varying CD19 expression on B cells. Higher CD19 expression leads to additional binding to CAR T-cells and subsequent activation, increasing CAR T-cell expansion. This looks different for each of the patient parameterizations; simulated CK for patient F0110 shows greater sensitivity to CD19 expression compared to that of patient F0104. For F0104, higher CD19 expression leads to faster expansion and faster contraction, causing a sharper peak in the CAR T CK. Higher CD19 expression also leads to greater long-term persistence of CAR T-cells. Patient F0110 exhibits greater expansion and persistence with varying levels of CD19 expression, with no evident contraction phase.

Examining the individually fit parameters for F0104 and F0110 sheds light on the unique behaviors of both the CAR T-cells and tumor cells between patient simulations. Patient F0104 has a smaller fraction of effector CAR T-cells that become memory cells, a larger initial tumor burden, and a slightly higher number of CAR T-cell divisions upon activation compared to patient F0110. This leads to greater expansion (and therefore greater efficacy) of the CAR T-cells for patient F0104, but potentially less persistence. For patient F0110, the lower expansion and greater memory cell formation leads to no clear contraction phase in the CK. The corresponding tumor growth curves show no impact of treatment except a small reduction in tumor growth rate at the highest receptor expression level scanned.

**FIGURE 5:**
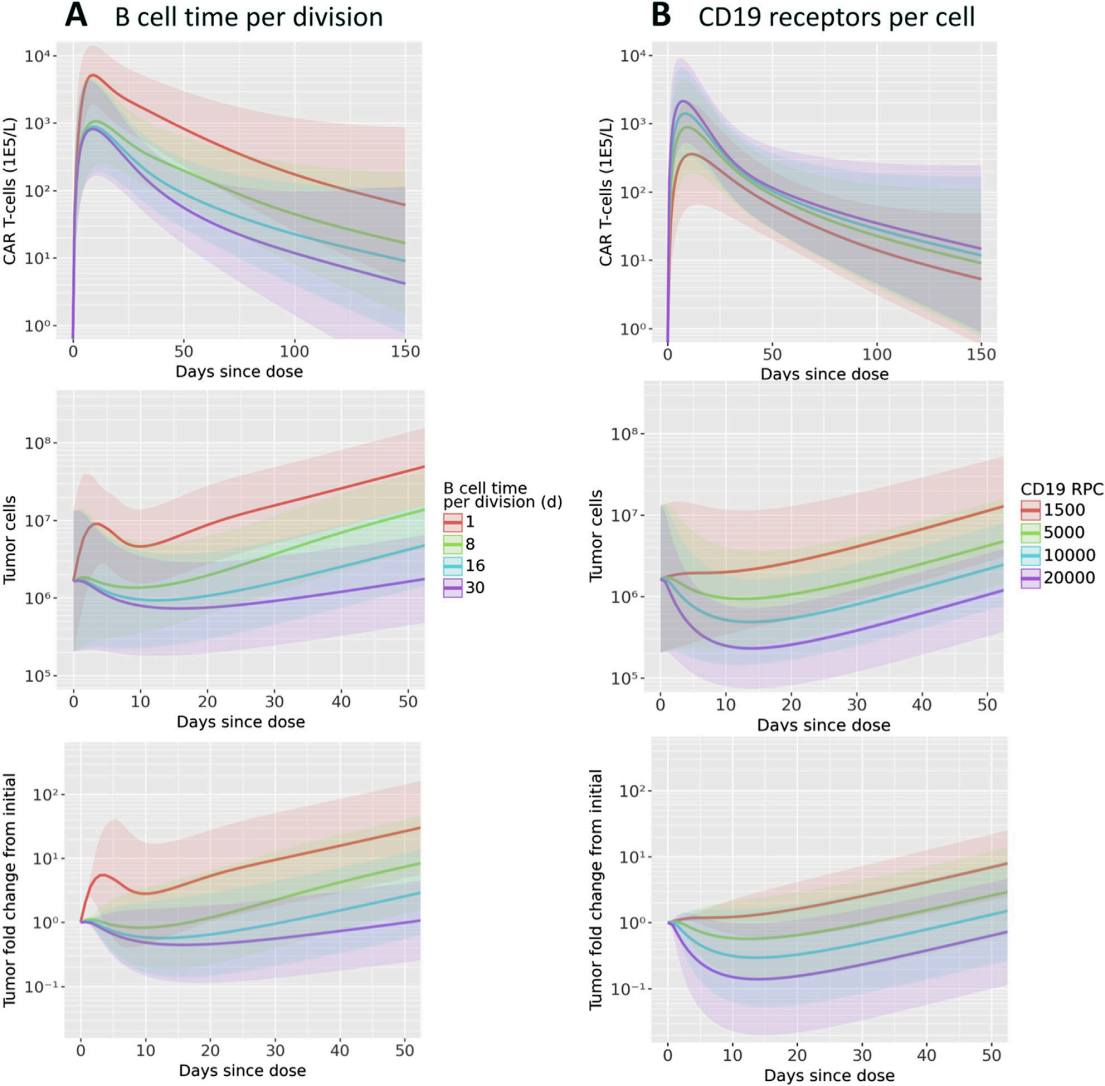
Results of scanning key model parameters representing patient characteristics across all patient parameterizations. Parameters were scanned across ranges consistent with values reported in the literature. Simulations of CAR T-cell concentration, total B cells, and B cell fold change from initial are shown for scans of **(A)** B cell time per division and **(B)** CD19 receptors per cell. Mean and one standard deviation of all patient results are shown.

To assess the behavior of CAR T-cells and tumor growth on a population level, the parameter value for either B cell division time or CD19 expression was updated one at a time for each patient. These parameters were varied ranges described in literature: Roesch et al. (2014) report NHL doubling times from 24 h to 30 days, and D’Arena et al. (2000) report a standard deviation for CD19 expression of about 1/3 the mean value. The minimum value for CD19 expression reported was much lower (10-fold lower than the mean value), which we also include in the parameter scan. Figure 5 shows the mean and standard deviation across all patients for CK, tumor cell count, and tumor fold change. Overall, the same patterns described above in the patient-specific scans hold true: faster B cell division times yield more CAR T-cell expansion and greater tumor growth, and higher CD19 expression leads to more CAR T-cells and improved tumor cell killing. Within the physiological ranges tested, B cell division time has an impact on both CK and tumor cell growth by close to an order of magnitude, on average. Notably, the rate of tumor regrowth is similar for all tumor doubling times, indicating that the increased persistence of CAR T-cells does counteract the increased tumor growth.

The range of reported CD19 expression is quite varied, and the model predicts that this parameter could have a significant impact on treatment efficacy. Between the maximum and minimum values scanned, within the range of reported values, there is about an order of magnitude difference in the CAR T cell Cmax. Furthermore, for the lowest CD19 RPC, there is essentially no tumor growth inhibition. The three higher RPC values do show inhibition, with a reduction from baseline of up to 10x.

### 3.4 Exploratory analysis: memory cell killing

**FIGURE 6:**
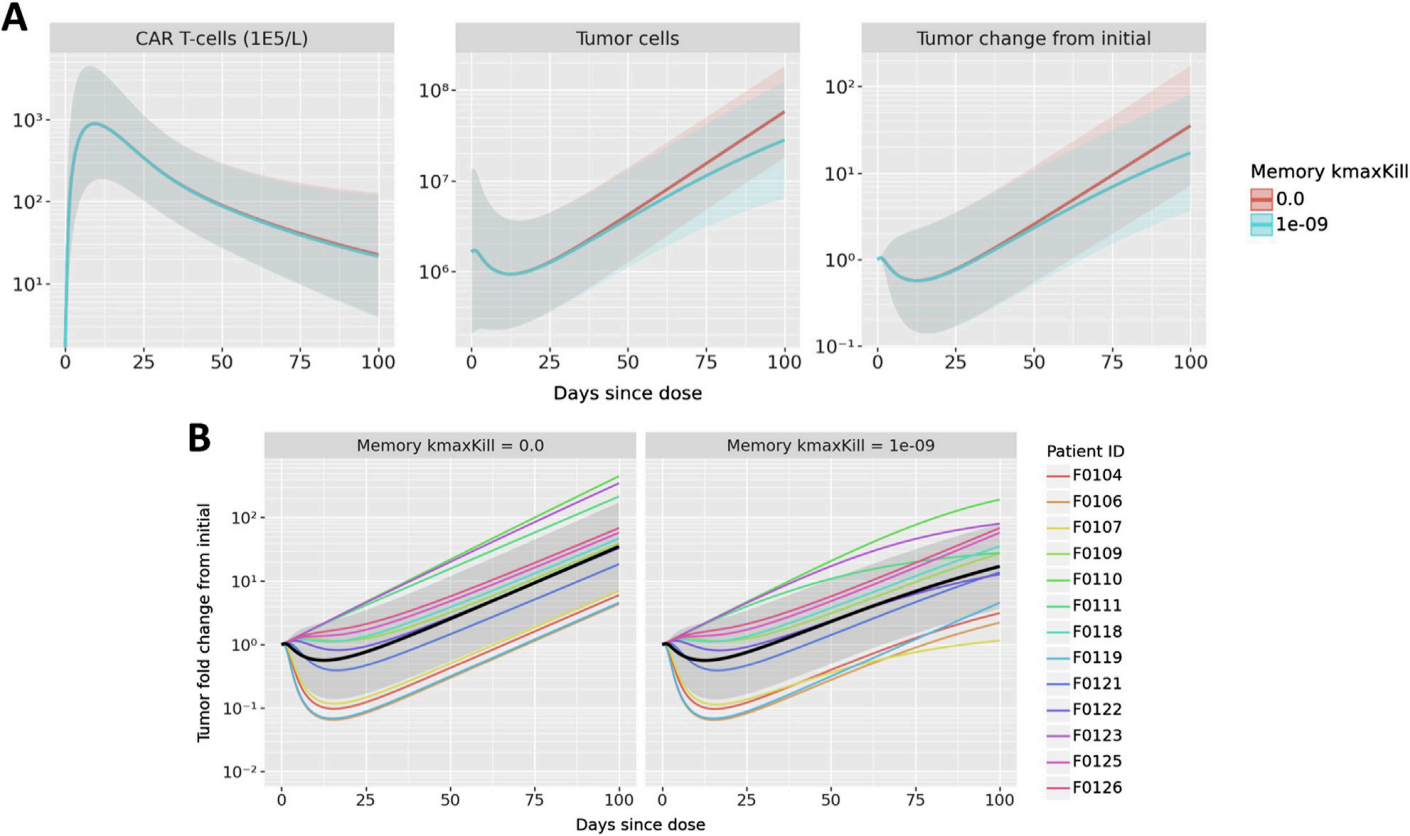
Results of exploring the impact of memory cell killing. Simulations in which memory cells have the same killing capacity as active cells are compared to the nominal case in which memory cells do not kill tumor cells. Simulations of CAR T-cell concentration, total B cells, and B cell fold change from initial are shown in **(A)** where mean and one standard deviation of all patient results are compared. B cell fold change from initial is shown in **(B)**; multicolored lines represent individual patient trajectories, black curve represents the mean across all patients, and the gray region represents one standard deviation.

To show how the model can explore questions about both individual and population-level dynamics, we performed simulations to understand the potential impact of memory cell killing. In the nominal simulations, we assume that memory cells do not kill tumor cells. For this analysis, we compare the nominal simulations against those in which memory cell killing has the same killing capacity as activated cells. Figure 6 shows the results for both the population level and individual trajectories.

First observing the population-level dynamics in Figure 6A, the impact on memory cell killing is observed only in the tumor, not in CAR T CK. Furthermore, the model predicts that any difference is observed after 50 days. This makes sense due to the delayed appearance of memory cells and the subsequent growth of the memory cell population - thus, memory cell killing is predicted to have a small overall impact on reducing tumor growth during the terminal phase of CAR T expansion. Since the exact memory cell populations may vary from patient to patient, the impact of memory cell killing may also be observed on a patient level, shown in Figure 6B. It is evident that some patients show little impact of memory cell killing, sch as F0125 and F0126. On the other hand, tumor growth in patients F0107, F0111, F0123 nearly plateaus as compared to the nominal parameterization which has linear growth.

Although there is insufficient data to inform the true activity of memory CAR-T cells in the model, this hypothetical analysis shows the ability of the model to differentiate the impact of treatment on individual patients as compared to a population-level impact. In these simulations, a moderate population-level effect was the result of an aggregated variety of patient effects, from no impact to a signficiant impact. Furthermore, the model shows in what populations and at what times the impact of these changes might be observed.

## 4 Discussion

Our mechanistic modeling approach incorporating molecular-scale and cell-scale dynamics successfully captured CAR T CK-PD and revealed key system behaviors. Mechanistic modeling is necessary to capture the interplay of target engagement, T cell expansion, and tumor cell killing. CAR T-cell therapy is distinct from other therapeutics in that CK and PD are inter-dependent. This dependency is demonstrated in our model by the sensitivity of Cmax to tumor and binding parameters.

Global sensitivity analysis revealed that both drug-specific and patient-specific properties can potentially explain variability in response to CAR T therapy. The most influential drug-specific properties are the number of divisions per activated CAR T-cell and the binding of the CAR for CD19. The number of divisions for activated cells is the most influential factor for peak CAR T-cell expansion, and was also highly influential for tumor killing. This number of divisions could potentially be increased through further engineering or refining of manufacturing processes for the CAR T product, for example, through selection for naive cells (Arcangeli et al., 2022). Importantly, while we classify this as a drug-specific property, this could be variable across patients since the CAR T-cells are manufactured from the patients’ own cells. Thus, individual variability in the number of T cell divisions can also contribute to observed variability in CK and efficacy. Binding affinity also impacted both CAR T-cell expansion and efficacy, which could be improved in engineering of the CAR.

While our modeling suggests that CAR T expansion is driven primarily by number of divisions, global sensitivity analysis shows that tumor properties such as CD19 expression and growth rate are comparatively more influential in driving efficacy. Tumor growth rate was also highly influential on CK. This demonstrates two things: (1) while expansion often correlates with efficacy, expansion itself is not necessarily sufficient for tumor shrinkage, and (2) variability in patient characteristics will lead to significant variability in both exposure and response. Modeling provides insight into this variability and can be used to inform patient, target, and indication selection.

In individual- and population-level model simulations, we observed that although a faster tumor growth rate corresponds to increased CAR T-cell expansion and distinct CK profiles, this increased expansion is often not enough to control the faster-growing tumor. This implies that drug characteristics may need to be modified in order to target more aggressive tumors. Notably, while there is little to no predicted tumor shrinkage for this CAR T with faster growing tumors for most patients, treatment is still effective in slowing tumor growth both short- and long-term, providing a benefit to patients. Increased target expression drives both increased expansion and stronger tumor killing. Patients with low target expression may be poor candidates for this type of treatment due to poor expansion and little anti-tumor activity, leading to lack of response. This also suggests that target expression should be a key consideration in both target and indication selection, while balancing toxicity concerns. Furthermore, the power of individualized parameterizations of the model was demonstrated in the memory cell killing exploratory analysis. Although the population-level simulation showed a small overall reduction in tumor growth, some patient trajectories showed signficant reduction while others showed nearly no impact. Although this was a hypothetical exploration due insufficient data, these simulations demonstrate that modeling can have a large impact on understanding individual patient dynamics.

In the future, this model and analysis could help drive decisions in CAR T-cell design, manufacturing, patient selection, patient-specific dose selection, and efficacious dose selection for novel CAR Ts. This model could be further refined by adding other T cell phenotypes, cytokines, immune cells types, and additional reactions such as re-activation of memory cells. One key limitation of the current work is lack of direct measurements of tumor burden over time to inform efficacy. Rather, we relied of B cell aplasia data and assumptions about the native immune population to estimate tumor reduction. Additional efficacy data would help to better constrain the model and may allow for individualized efficacy modeling. Additional patient-specific data such as CD19 expression could enable individualized predictions of efficacy/response through a digital twin approach. Another limitation of this model is that it does not account for effects of CD4^+^ T cells on tumor cell killing. This model could also be extended to study other targets and indications, including solid tumors for which there are currently no approved CAR T-cell therapies.

## Data Availability

Publicly available datasets were analyzed in this study. This data can be found here: https://www.ncbi.nlm.nih.gov/pmc/articles/PMC7518381/.
